# Does the Hirsch Index Improve Research Quality in the Field of Biomaterials? A New Perspective in the Biomedical Research Field

**DOI:** 10.3390/ma11101967

**Published:** 2018-10-13

**Authors:** Saverio Affatato, Massimiliano Merola

**Affiliations:** Laboratorio di Tecnologia Medica, IRCCS-Istituto Ortopedico Rizzoli, Via di Barbiano, 1/10 40136 Bologna, Italy; massimiliano.merola@tecno.ior.it

**Keywords:** h-index, bibliometric indicators, biomaterials, quality of research, citations

## Abstract

Orthopaedic implants offer valuable solutions to many pathologies of bones and joints. The research in this field is driven by the aim of realizing durable and biocompatible devices; therefore, great effort is spent on material analysis and characterization. As a demonstration of the importance assumed by tribology in material devices, wear and friction are two of the main topics of investigation for joint prostheses. Research is led and supported by public institutions, whether universities or research centers, based on the laboratories’ outputs. Performance criteria assessing an author’s impact on research contribute somewhat to author inflation per publication. The need to measure the research activity of an institution is an essential goal and this leads to the development of indicators capable of giving a rating to the publication that disseminates them. The main purpose of this work was to observe the variation of the Hirsch Index (h-index) when the position of the authors is considered. To this end, we conducted an analysis evaluating the h-index by excluding the intermediate positions. We found that the higher the h value, the larger the divergence between this value and the corrected one. The correction relies on excluding publications for which the author does not have a relevant position. We propose considering the authorship order in a publication in order to obtain more information on the impact that authors have on their research field. We suggest giving the users of researcher registers (e.g., Scopus, Google Scholar) the possibility to exclude from the h-index evaluation the objects of research where the scientist has a marginal position.

## 1. Introduction

Joint replacement surgery is a successful and consolidated branch of orthopaedics. Its progressive achievement in alleviating pain and disability, helping patients to return to an active life, is reliant on efficient relationships between clinicians and researchers working across transverse areas of medicine and science [1]. The purpose of tribology research applied to orthopaedics is the minimization and elimination of losses resulting from friction and wear [2]. The research of new biomaterials plays an important role, and as a consequence, in vitro tests for such materials are of great importance [3]. The knowledge of the laboratory wear rate is an important aspect in the preclinical validation of prostheses. Research and development of wear-resistant materials continues to be a high priority [4,5,6]. Clinical research designed to carefully evaluate the performance of new materials intended to reduce wear is essential to ascertaining their efficacy and preventing the possibility of unexpected failure [7,8]. Unfortunately, failures and revision surgeries still constitute the main clinical problems related to total joint replacement [9]. The research is therefore constantly pushed to find new solutions to wear-related issues and to identify new high-standard materials. The public institutions of research receive national funding on the bases of their results and are therefore constrained to obtain high levels of quality assessment [10]. Evaluation of scientific publications is the criterion used by the universities and research institutes to measure the merit and value of researchers and academics [11], and it has a crucial impact on research funds distribution [12,13,14]. The need to measure the research quality of institutions is an essential goal, which has led to the development of indicators capable of giving a rating to publications. These indicators are used in bibliometric disciplines to quantitatively evaluate the quality and diffusion of scientific production within the scientific community. In order to obtain funds within the orthopaedic community, there is a strong pressure on researchers to publish even if the merit of the study is unreliable; this is because objectives are set to achieve a certain number of publications instead of focusing on the quality of the research [15]. 

There are two main ways to evaluate scientific research:

- Bibliometric indicators are quantitative methods based on the number of times a publication is cited. The higher the number of citations, the larger the group of researchers who have used this work as a reference and, thus, the stronger its impact on the scientific community;
- Peer review is a qualitative method based on the judgement of experts. A small number of researchers, specialized in the field of the work, analyze and evaluate the scientific value of a publication.

Eugene Garfield proposed the Impact Factor (IF) in 1955 [16,17] with the intent to help scientists look for bibliographic references; the IF indicator was quickly adopted to assess the influence of journals and, not long after, of individual scientists. A journal’s impact factor is the ratio of two elements: the numerator is the number of citations in the current year to items published in the previous two years, and the denominator is the total number of articles published in the same two years [16,17]. It is published by the ISI-Thomson publisher on the basis of the Web of Science database and measures the frequency with which an article published in a journal is cited by other periodicals over a specific period of time (two years after its release). This measure is used as an appraisal of the importance of a magazine compared with others in the same sector: the higher the impact factor, the more authoritative the magazine [18]. The impact factors of each magazine can be consulted on the website of the Journal of Citation Reports (JCR) [19].

The impact factor is widely used as an index of academic research quality. It is also applied as a winning criterion for the granting of funds and incentives, or as a basis for the evaluation of a scholar or a professional in public competitions [11]. Yet the impact factor is an indicator for measuring the impact of a magazine in its specific disciplinary area, certainly not for evaluating the authors, and this latter use has been criticized for many reasons. In order to overcome the problems of the impact factor, in 2005, Jorge E. Hirsch [20] proposed a new index, known as the h-index or Hirsch index, as a single-number criterion to evaluate the scientific output of a researcher. It combines a measure of quantity (publications) and impact (citations). In other words, a scientist has index h if h-many of his articles have at least h-many citations each, and the other articles have fewer than h-many citations each [21]. It performs better than other single-number criteria previously used to evaluate the scientific output of a researcher (impact factor, total number of documents, total number of citations, citation per paper rate, and number of highly cited papers) [22]. The h-index is easy to understand and can be easily gained by anyone with access to the Thomson ISI Web of Science [22]. Actually, the h-index has been reviewed as one of the most reliable criteria for evaluating the scientific outputs of researchers [23]. This index has many flaws: the articles with citations less than the h-index value are excluded from the calculation. The number of citations is influenced by self-citations and colleague citations, meaning that its value can be increased by recommendation to friends and colleagues [23,24,25]. There are many circumstances where the h-index provides misleading information on the impact of an author. However, this popular bibliometric indicator does not consider multiple co-authorship nor author position [11,26]. To our knowledge, no policy guides author order in biomedical publications. The position of an author, controversies about author order, and disagreements on the involvement of the last author are constantly debated; thus, it is worth analyzing the relationship between author position and bibliometric indicators [27]. The contribution of an author to a research project is not always clear, especially when a manuscript is attributed to a large group [28].

Rating and weighting of research value is not an easy task, as history shows; often, what is believed to be modern and mainstream research will yield highly rated papers but is not always a guarantee of innovation and scientific progress [15]. With this in mind, and to go more into depth in this matter, we produced a statistical evaluation of the h-index on a cohort of 60 authors belonging to the biomedical field, accounting for only given positions. In detail, three modified h-values were evaluated: considering only the First (F) and Second (S) authorships (referred to as FS); only the F and the Last (L) authorships (referred to as FL); and the F, S, and L positions (referred to as FSL). The main goal of this work was to implement an algorithm that can calculate a modified h-index considering the author’s position in an article. 

Different approaches are available to decide the author order, like sorting them alphabetically or listing them in descending order according to their contribution [29]. Several approaches may be used to assess the contribution of an author to a paper [30]. In the “sequence-determines-credit” (SDC) system, the author order reflects the declining importance of their contribution. The “equal contribution” approach uses alphabetical order and implies identical involvement. The “first-last-emphasis” norm underlines the importance of the last author. Using the “percent-contribution-indicated” implies detailing each author’s impact. 

The convention used in the biomedical field of research, as reported in the literature [27], is as follows: the first author conducts the majority of the work; the last author could be the senior member of the group and usually leads the research; co-authors, those between the first and last, are ranked in order of their input to the work; the corresponding authors—typically senior scholars—communicate with editors and readers. With this premise, we considered the first, the second, and the last authors as the main contributors to biomedical research.

## 2. Methods 

A total of 60 authors from the biomedical field were selected as a cohort; 30 of them were obtained from an Italian scientist ranking system [31] and the other 30 were chosen from all around the world.

**Figure 1:**
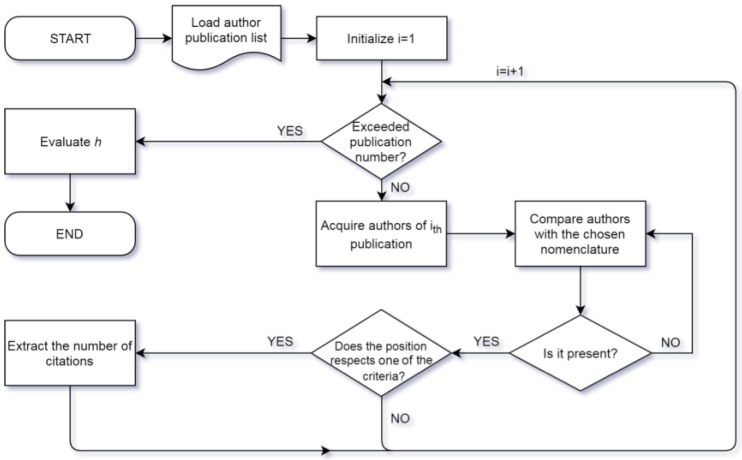
Flowchart of the process to obtain the corrected h-value.

To obtain the modified h-index, an algorithm was implemented in MATLAB (MathWorks, Natick, MA, USA). The complete list of articles attributed to an author was extracted from the scopus.com webpage using the implemented feature called “Export all”. The information extracted was limited to Authors, Title, and Citation Count. Each list was obtained in the csv extension; these lists were then imported into the workspace of MATLAB using the Import Tool App. Each author entry was analyzed to find out their position; thus, according to the excluding criterion, the publication was considered or not for the evaluation of h. Given that authors are frequently cited in different ways (e.g., Surname, Name; Surname, N.; Surname M.N., etc.), the investigation considered all different options. If a publication respected the inclusion criterion, its citation count was taken into account. The list of included citation counts was sorted in ascending order; thus, the h value was by evaluated considering the last position at which the citation count was higher than or equal to the position itself. In Figure 1, a flow chart of the exclusion process is shown. 

## 3. Results

**Figure 2:**
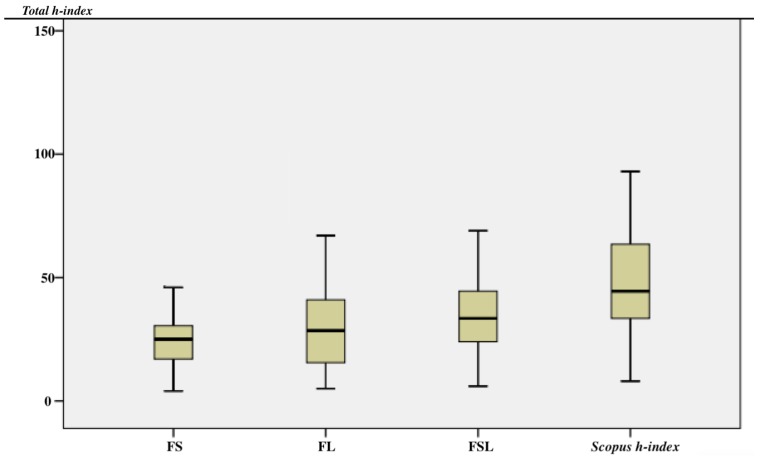
A box plot shows the modified h-index values (± standard deviation) for all authors considered in this study. The total h-index retrieved from Scopus is the highest of the four classifications.

In Figure 2 are summarized the results obtained through the different exclusion criteria here studied. It is worth underlining the large divergences obtained comparing the higher h-values with their respective corrected ones.

From the results in Figure 2, we emphasize that on the basis of our exclusion criteria, the modified h-value decreases significantly.

**Figure 3:**
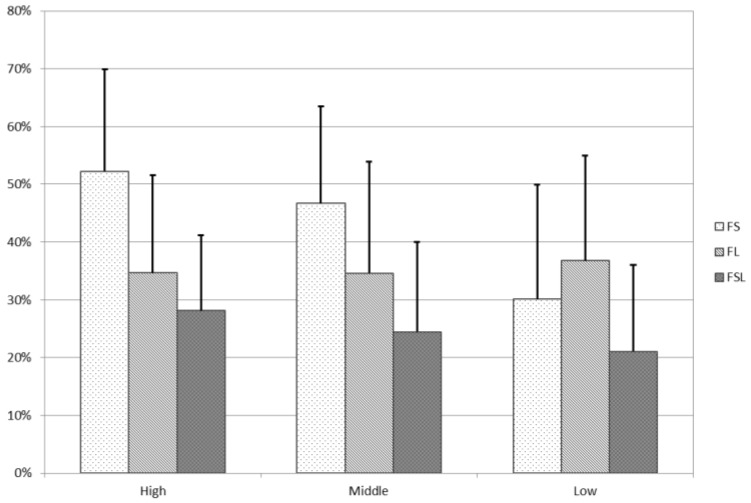
Histogram of the influence of the exclusion criteria on the h-values in sub-cohorts based on the starting h from Scopus.

To highlight these differences, the entire cohort was divided into three sub-cohorts. A first group of authors with h-value ranging from 0 to 35 (called Low), a second group from 36 to 50 (Middle), and a third group from 51 up to the maximum of 181 (High) were extracted, as shown in Figure 3.

In Figure 3 it is underlined that the High sub-cohort, starting from the highest value of h, is the most affected by all the exclusion criteria. This is especially true in the FS case, where it reaches a mean reduction of more than 50%. On the contrary, for the Low group, this exclusion criterion only decreases the h-value by roughly 30%. The Low group is more influenced by the exclusion of the publications where the authors are present as second authors (35% of decrease). The Middle group is more affected by the FS criterion, followed by the FL and the FSL.

**Figure 4:**
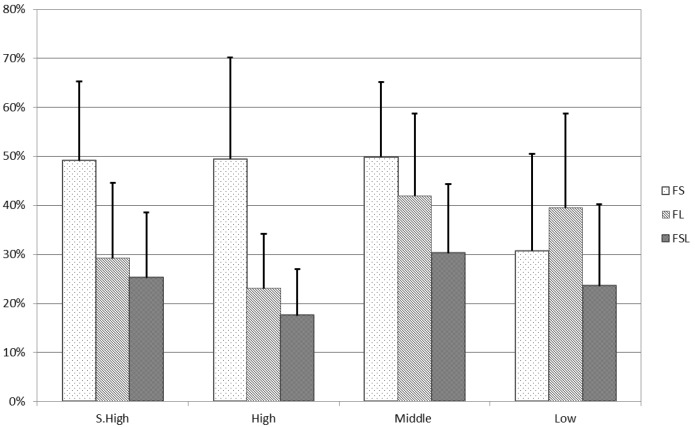
Histogram of the influence of the exclusion criteria on the h-values with sub-cohorts based on the number of publications.

In Figure 4, another histogram of the influence of the exclusion criteria is presented. In this case, the sub-cohorts were obtained based on the number of articles each author has. Considering the large range (from a minimum of 15 to a maximum of 1241), we chose to obtain a further group, yielding a total of four. The Low group collected the authors with up to 150 publications, the Middle group ranged from 151 to 300, High from 301 to 500, and Super High (S. High) from 501 to the maximum.

This representation also outlined how the authors with a great number of publications are more affected by the exclusion criteria that do not take into consideration the articles where they are listed last among the authors. The first three sub-cohorts have a mean reduction of around 50% from their starting h-value when the FS criterion is applied. On the counter side, the FL criterion more greatly affects the Middle and the Low cohorts, reaching roughly 40% against the 30% and 20% of the Super High and High, respectively. The FSL criterion has a similar outcome on the group, but is stronger on the Middle and the Super High sub-cohorts, where it is about 30%.

## 4. Discussion and Conclusions

The term “impact factor” has gradually evolved to describe both journals’ and authors’ impacts. Journal impact factors generally involve relatively large populations of articles and citations. There are some ambiguities in the use of the h-index that could provoke changes in the publishing behaviour of scientists, such as increasing the number of self-citations distributed among the documents on the edge of the h-index [32]. Another disadvantage of the h-index is that not all citations of a researcher are involved in the calculation and its value may not increase with a rise in citations. Highly cited papers are significant for the evaluation of the h-index, but once they belong to the top h-many papers, the effect of number of citations they get is negligible [22]. Considering that the evaluators reduce scientific success to a single value, researchers can change their behaviour to increase these values, even by using unethical strategies [33]. Moreover, the scientific independent criterion of peer-review evaluation is going to be replaced by a system of private companies whose only feedback is the indicator number [11]. The authors believe that it is essential to continue analyzing the bibliometric indicators in order to establish their drawbacks and limitations and to propose improvements where necessary. It is especially relevant to determine in which cases this index could be biased, since it could have serious consequences for the assessment of scientists and academics. Our work proposes to be an insight into the bibliometric indicators and to help the scientific community and research institutions consider how authorship position impacts on the h-value.

The main purpose of this work was to observe the variation of the h-value when the position of authorship is considered. Therefore, an empirical analysis was conducted to assess the influence of an author’s intermediate positions on their h-index. The results of this study showed that when the h-value is high, there is a large divergence between this value and the “corrected” one. We propose an improved method to consider the importance of the authorship in a publication in order to obtain a more profound understanding of the effective impact that an author has on their research field. This could be realized by giving users of researcher registers (e.g., Scopus, Google Scholar, etc.) the possibility to select which version of the h-index they want to analyze. The users should have the possibility, along with the already-implemented exclusion of self-citations, to exclude the publications where the authors have an intermediate position. It is worth noting that this correction should be considered for senior researchers, whereas young ones often have difficulties obtaining a relevant position in authorship. Thus, this exclusion can negatively affect the process of researcher renewal, discouraging young ones who would not see their efforts rewarded.

We believe that bibliometric indices should evolve with the aim of fairer evaluation of scientific productions. This would also be beneficial for funding distribution in the biomedical and orthopaedic research spheres, above all considering the threat of research cuts being to the detriment of patients’ health. Alternatively, there is the risk that research toward new and more efficient biomaterials could stagnate due to a lack of capital granted to deserving scientists.
